# Monoamine Oxidase-Dependent Pro-Survival Signaling in Diabetic Hearts Is Mediated by miRNAs

**DOI:** 10.3390/cells11172697

**Published:** 2022-08-30

**Authors:** Stefano Cagnin, Marco Brugnaro, Caterina Millino, Beniamina Pacchioni, Carmen Troiano, Moises Di Sante, Nina Kaludercic

**Affiliations:** 1 Department of Biology, University of Padova, 35131 Padova, Italy; 2 CIR-Myo Myology Center, University of Padova, 35131 Padova, Italy; 3 Department of Biomedical Sciences, University of Padova, 35131 Padova, Italy; 4 Neuroscience Institute, National Research Council of Italy (CNR), 35131 Padova, Italy; 5 Fondazione Istituto di Ricerca Pediatrica Città della Speranza (IRP), 35127 Padova, Italy

**Keywords:** diabetic cardiomyopathy, autophagy, monoamine oxidase, miRNAs, pro-survival pathways

## Abstract

Diabetes leads to cardiomyopathy and heart failure, the leading cause of death for diabetic patients. Monoamine oxidase (MAO) inhibition in diabetic cardiomyopathy prevents oxidative stress, mitochondrial and endoplasmic reticulum stress and the development of diastolic dysfunction. However, it is unclear whether, in addition to the direct effects exerted on the mitochondria, MAO activity is able to post-transcriptionally regulate cardiomyocyte function and survival in diabetes. To this aim, we performed gene and miRNA expression profiling in cardiac tissue from streptozotocin-treated mice (model of type 1 diabetes (T1D)), administered with either vehicle or MAOs inhibitor pargyline for 12 weeks. We found that inhibition of MAO activity in T1D hearts leads to profound transcriptomic changes, affecting autophagy and pro-survival pathways activation. MAO activity in T1D hearts increased miR-133a-3p, -193a-3p and -27a-3p expression. These miRNAs target insulin-like growth factor receptor 1 (*Igf1r*), growth factor receptor bound protein 10 and inositol polyphosphate 4 phosphatase type 1A, respectively, all components of the IGF1R/PI3K/AKT signaling pathway. Indeed, AKT activation was significantly downregulated in T1D hearts, whereas MAO inhibition restored the activation of this pro-survival pathway. The present study provides an important link between MAO activity, transcriptomic changes and activation of pro-survival signaling and autophagy in diabetic cardiomyopathy.

## 1. Introduction

There is a higher incidence of cardiovascular events in diabetic patients. Diabetes is a risk factor for left ventricle (LV) dysfunction, also called diabetic cardiomyopathy, and is the most common, disabling and deadly of diabetic complications. Mitochondrial bioenergetics is impaired in diabetic patients and, together with oxidative stress, contributes to the development of cardiovascular complications. In addition to cytoplasmic sources of reactive oxygen species (ROS), such as NADPH oxidase, shown to contribute to diabetes, it is widely accepted that the majority of ROS arise from the mitochondria. For instance, mitochondrial respiratory chain-generated superoxide is considered the key event in the activation of all the other pathways involved in the pathogenesis of diabetic complications [1]. In addition to this, other mitochondrial proteins play a major role in the oxidative stress and development of several cardiovascular pathologies. Genetic deletion of p66^Shc^ dramatically reduces ROS formation in experimental models of atherosclerosis, diabetes and ischemia/reperfusion injury [2,3,4]. NOX4, at the interface between mitochondria and ER, also contributes to the development of LV dysfunction in several models of heart failure [5,6,7]. In addition to those enzymatic systems, mitochondria also contain monoamine oxidases (MAOs), whose role in terminating neurotransmitter signaling in the brain is well established, whereas its modulation of cardiac morphology and function is still being elucidated [8,9].

Monoamine oxidases (MAOs) are mitochondrial flavoenzymes responsible for biogenic amine catabolism resulting in hydrogen peroxide (H_2_O_2_) formation. We and others previously described a direct effect of MAO-dependent ROS formation on mitochondrial and cardiomyocyte function in several models of heart failure [4,10,11,12,13,14], including diabetic cardiomyopathy [15,16]. Indeed, we demonstrated that MAOs are the major source of mitochondrial ROS in cardiomyocytes exposed to high glucose and pro-inflammatory cytokine IL-1β and that MAO-dependent ROS formation is able to directly induce mitochondrial dysfunction by promoting the opening of the mitochondrial permeability transition pore [15]. Importantly, MAO-dependent mitochondrial ROS formation and organelle dysfunction acted upstream of endoplasmic reticulum (ER) stress, a well-known pathogenetic factor that contributes to the development of diabetes-associated complications. Pharmacological inhibition of MAO activity in vivo in a mouse model of type 1 diabetes (T1D) prevented diastolic dysfunction, oxidative and ER stress, as well as remodeling of the extracellular matrix and development of fibrosis [15].

H_2_O_2_ is able to modulate the expression of different microRNAs (miRNAs) in the cardiovascular system [17,18], indicating that they may play a role in cardiac diseases related to ROS. MiRNAs are a class of small noncoding RNAs (18–22 nucleotides) encoded by plants and the metazoan genome that regulate mRNA stability and protein expression. MiRNAs play a role as regulators of gene expression by binding to complementary sequences on the 3′-untranslated region (3′-UTR) of their target mRNA, thus inhibiting mRNA translation or promoting mRNA degradation [19]. A single miRNA can regulate several hundreds of transcripts, making it possible to switch instantly between cellular programs. Therefore, they are often viewed as master regulators of multiple protein-coding genes. A number of studies highlighted the key role of miRNAs in maintaining proper physiological properties of the cardiovascular system, as well as participating in pathological processes [17,20,21,22,23,24,25]. An increasing number of studies show that intracellular ROS can either inhibit or induce miRNA expression levels, which leads to subsequent biological effects through the regulation of their direct target genes [19]. Moreover, position-specific oxidation of miRNAs was shown to serve as an epi-transcriptional mechanism that coordinates pathophysiological redox-mediated gene expression [26]. Nevertheless, it is currently unknown whether MAO’s activity controls the expression of certain miRNAs and whether MAO-dependent dysregulation of miRNAs occurs in pathological conditions. 

In this work, we tested whether, in addition to the direct effects exerted by MAO-derived H_2_O_2_ on mitochondria, MAO activity is able to post-transcriptionally regulate cell function and survival in pathological conditions. Therefore, we performed gene and miRNA expression profiling in cardiac tissue from a mouse model of T1D with or without pharmacological MAO inhibition. The transcriptomic analysis highlighted that activation of catabolic processes, such as autophagy, is likely one of the mechanisms involved in disease development promoted by MAO, leading to mitochondrial and cellular derangements in diabetic conditions. Moreover, we identified a specific subset of miRNAs modulated by MAO activity in diabetic hearts that are involved in pro-survival signaling.

## 2. Materials and Methods

### 2.1. Animal Model of T1D

All the animal studies were performed using male C57BL6/J mice (6–7 weeks of age and at least 20 g in weight; Charles River Laboratories, Calco, Italy). Five mice per cage were housed in the animal facility of the University of Padova with 12 light/12 dark cycles. T1D was induced with streptozotocin (50 mg/kg/day in citrate buffer pH 4.5) administered intraperitoneally for five consecutive days [15]. Streptozotocin is a glucosamine–nitrosourea compound toxic to pancreatic β-cells. Administration of multiple low doses of streptozotocin was chosen due to reduced acute toxicity of the compound, resulting in low acute mortality (< 5%). Mice were then randomized to receive either vehicle or MAO inhibitor pargyline (50 mg/kg/day via intraperitoneal injection) for the whole duration of the experimental protocol (i.e., 12 weeks). Blood glucose levels were measured twice a month using a glucose meter (OneTouch Ultra 2, Lifescan, Malvern, PA, USA), and mice with blood glucose levels ≥ 17 mM were considered diabetic. The following groups were examined: (i) control mice (C), (ii) streptozotocin-treated mice (D), (iii) control mice treated with pargyline (C + P) and (iv) streptozotocin mice treated with pargyline (D + P). All aspects of animal care and experimentation were carried out in compliance with European and Italian (D.L. 26/2014) laws concerning the care and use of laboratory animals and were approved by the local ethical committee of the University of Padova (Organism for Animal Welfare, OPBA) and by the Italian Ministry of Health (for ethical approvals, please see the Institutional Review Board Statement section).

### 2.2. Cell Culture and miRNA Transfection

Neonatal rat ventricular myocytes (NRVMs) were isolated from 1 to 3 days old rats as previously described [12,13] and plated at the density of 400,000 cells/mL in minimum essential media (MEM) supplemented with FBS 10%, 0.1 mM BrdU, antibiotics and non-essential amino acids. Cells were maintained at 37 °C with 5% CO_2_. The medium was changed to MEM with 5 mM glucose, supplemented with 1% FBS after 24 h. For the experiments, cells were cultured either with high mannitol (20 mM) as an osmotic control or high glucose (20 mM). In order to block the autophagy flux, cells were incubated with chloroquine (50 µM) for 4 h prior to cell harvesting. MAO-A was silenced as previously described [15].

HL-1 cardiomyocytes (Sigma-Aldrich, St. Louis, MO, USA) were cultured in 24 well plates using DMEM/F12 (Thermo Fisher Scientific, Waltham, MA, USA) containing 2 mM L-Glutamine (EMD Millipore, Burlington, MA, USA), 12.5% FBS (EMD Millipore, Burlington, MA, USA) and 1X Penicillin-Streptomycin Solution (Thermo Fisher Scientific, Waltham, MA, USA). Cells were divided before reaching 95% of confluence. An amount of 10 μM of miR-27a-3p or -193a-3p mimic was transfected using Lipofectamine 2000 (Thermo Fisher Scientific, Waltham, MA, USA), as previously described [27]. Cells transfected with miRNA scramble were used as controls. RNA was extracted 48 h after transfection and used to test the expression of target genes using qRT-PCR.

### 2.3. RNA Extraction

TRIzol Reagent (Thermo Fisher Scientific, Waltham, MA, USA) was used to extract RNA from heart samples (1 mL/30 mg of tissue), primary cardiomyocytes and HL-1 cell cultures, as previously described [15,28]. Cell lysis was performed with TRIzol reagent (Thermo Fisher Scientific, Waltham, MA, USA) (500 μL per well in a 24-well plate) after one washing step with PBS, while tissue samples were lysed with TissueLyser II (QIAGEN) in 1000 μL of TRIzol (Thermo Fisher Scientific, Waltham, MA, USA). Chloroform (200 μL per 1000 μL of TRIzol) was added to the homogenate to separate the RNA-containing aqueous phase. Thereafter, RNA was precipitated by adding an equal volume of isopropanol to the aqueous phase. Qualitative and quantitative RNA measurements were assessed both by spectrophotometer and Agilent 2100 Bioanalyzer. RNA showing an RNA integrity number higher than 7 was used for experiments. 

### 2.4. Microarray Expression Profiles

SurePrint G3 Mouse Gene Expression 8 × 60 K Agilent chips were used to profile the expression of mRNAs. For the mRNA analysis, 200 ng of total RNA was used for Cy3 labeling with Low Input Quick Amp Labeling Kit (Agilent Technologies, Santa Clara, CA, USA) according to manufacturer instructions. The labeled sample was dispensed onto the microarray to perform hybridization at 65 °C for 17 h with 10 rpm rotation. At least three biological replicates were used for each condition (diabetes (D), control (C), diabetes treated with pargyline (D + P) and control treated with pargyline (C + P)).

Agilent Mouse miRNA Microarray 8  × 60 K platform (Agilent Technologies, Santa Clara, CA, USA) based on miRbase V.21 was used to profile the expression of miRNAs. An amount of 100 ng of total RNA was labeled using miRNA Complete Labeling and Hyb Kit (Agilent Technologies, Santa Clara, CA, USA), according to the manufacturer’s protocol. Labeled RNA was hybridized onto microarray slides using a rotational oven at 55 °C for 22 h. At least three biological replicates were used for each condition.

After hybridization, both mRNA and miRNA microarray slides were washed using the Wash Buffer Kit (Agilent Technologies, Santa Clara, CA, USA) and dried at room temperature. Microarray slides were scanned using a G2505C scanner (Agilent Technologies, Santa Clara, CA, USA) at 3 µm resolution. Probes features were extracted using the Feature Extraction Software v. 10.7.3.1 with GE 1 Sep09 protocol (Agilent Technologies, Santa Clara, CA, USA). 

### 2.5. Microarray Data Analyses

For the mRNA microarray, raw microarray gene expression data were quantile normalized, and probe expression values that did not pass the filter (positive and significant) were set as NA (not available). Probes with more than 50% of NA per condition were excluded from the analysis. Multiple *t*-tests were performed to identify differentially expressed genes, using adjusted Bonferroni correction and 0.05 as the *p*-value cut-off. Differentially expressed genes in a specific condition were identified by comparing gene symbols in Venn diagrams [https://bioinformatics.psb.ugent.be/webtools/Venn/, accessed on 26 August 2022]. In order to identify the effects of pargyline on diabetic hearts, differentially expressed genes were clustered using the Self Organizing Tree Algorithm (SOTA) method [29] with default parameters. Enrichment analysis of genes sharing the same expression profile was performed according to the DAVID web tool [30]. Gene Set Enrichment Analysis (GSEA) [31] was used to identify gene sets modulated by pargyline using KEGG and Wiki Pathways. All differentially expressed genes were used to calculate sample clustering using the Pearson correlation and a complete linkage. The identification of differentially expressed genes, SOTA and clustering analyses were performed using the TMev software [32]. 

Cytoscape V3.8 was used to represent the miRNA–target network [33]. The network was prepared considering predicted miRNA–target interactions and protein–protein interactions described in the BioGrid database V4.4.207 [https://thebiogrid.org/, accessed on 26 August 2022] for *Mus musculus*. For the miRNA microarray, raw microarray data were first filtered for the number of miRNAs presenting an expression value above the background (0% of undetected values were allowed for each miRNA) and then normalized according to the loess cyclic algorithm, as previously described [34]. The identification of differentially expressed miRNAs was performed as for mRNAs. MiRNAs and samples were clustered using average dot product with a complete linkage method implemented in the TMev software. MiRNA function was inferred using the TAM 2.0 tool with default parameters [35] and according to the function of predicted targets. In order to predict miRNA targets, five different algorithms were used: miRWalk [36], miRanda [37], miRDB [38,39], Targetscan [40] and TarBase [41]. Since bioinformatics algorithms may provide false positive results, we included biological evidence of possible interaction between miRNAs and their targets, calculating the expression correlation (Pearson correlation) and maintaining a miRNA–target relationship that showed a negative correlation. 

### 2.6. Quantitative Real-Time PCR Analysis (qRT-PCR)

cDNA reverse transcription was performed using reverse SuperScript IV (Thermo Fisher Scientific, Waltham, MA, USA). qRT-PCR was performed using Power SYBR Green PCR Master Mix (Applied Biosystems). Relative amounts of analyzed genes were calculated by the comparative ∆∆Ct method. The primers used are listed in Supplementary Table S1.

The TaqMan method was used to evaluate miRNA expression by qRT-PCR. An amount of 10 ng of total RNA was reverse transcribed using the TaqMan MicroRNA Reverse Transcription Kit (Thermo Fisher Scientific, Waltham, MA, USA), according to the user manual. qRT-PCR was performed with the 96 CFX System (BioRad, Hercules, CA, USA) in 20 μL using the TaqMan Universal PCR Master Mix II (Thermo Fisher Scientific, Waltham, MA, USA) according to the manufacturer’s protocol. PCR reaction was performed as follows: 50 °C for 2 min; 95 °C for 10 min; 95 °C for 15 s, 60 °C for 1 min, for 40 cycles. Data analysis was carried out according to the ∆Ct method using the U6 gene as a reference gene. Correlation between gene expression obtained with qRT-PCR and microarray was performed using Pearson correlation. 

### 2.7. Luciferase Assay

HL-1 cardiomyocytes were transfected with 100 pg/mL of pmirGLO Dual-Luciferase miRNA Target Expression Vector (Promega, Madison, WI, USA) containing the target sequence or a control sequence (primers for cloning are listed in Supplementary Table S2) and 10 μM of miRNA mimics. Assays were performed using the Dual-Luciferase Reporter Assay (Promega, Madison, WI, USA), measuring firefly and renilla luciferase activities with Turner Designs TD-20/20 Luminometer (DLReady, Promega, Madison, WI, USA). MiRNA transfections were independently replicated at least three times.

### 2.8. Western Blot

Primary cardiomyocytes or heart tissue were homogenized in a lysis buffer containing protease and phosphatase inhibitors. Protein concentration was determined using a BCA protein assay (Thermo Fisher Scientific, Waltham, MA, USA). Proteins were separated using SDS–PAGE and transferred to the nitrocellulose membrane (Thermo Fisher Scientific, Waltham, MA, USA). Following incubation with primary and secondary HRP-conjugated antibodies (Santa Cruz Biotechnology, Dallas, TX, USA, cat# sc-2357 or sc-516102), chemiluminescence was detected using UVITEC (Cambridge) and analyzed using ImageJ software. The following antibodies were used: cardiac actin (Santa Cruz Biotechnology, Dallas, TX, USA, cat# sc-58670), LC3B (Cell Signaling, Danvers, MA, USA, cat# 2775S), phospho-AKT Thr^308^ (Cell Signaling, Danvers, MA, USA, cat# 4056S), phospho-AKT Ser^473^ (Cell Signaling, Danvers, MA, USA, cat# 4060S) and pan-AKT (Cell Signaling, Danvers, MA, USA, cat# 4691S).

### 2.9. Statistical Analysis

All values are expressed as mean ± SEM unless otherwise stated in the figure caption. Comparison between groups was performed by one-way or two-way ANOVA, followed by Tukey’s post hoc multiple comparisons for normally distributed data and non-parametric Dunn’s test for not normally distributed data. Comparisons between two groups were performed using a non-paired two-tailed Student’s *t*-test. A value of *p* ≤ 0.05 was considered significant.

## 3. Results

### 3.1. mRNA Expression Analysis

**Figure 1:**
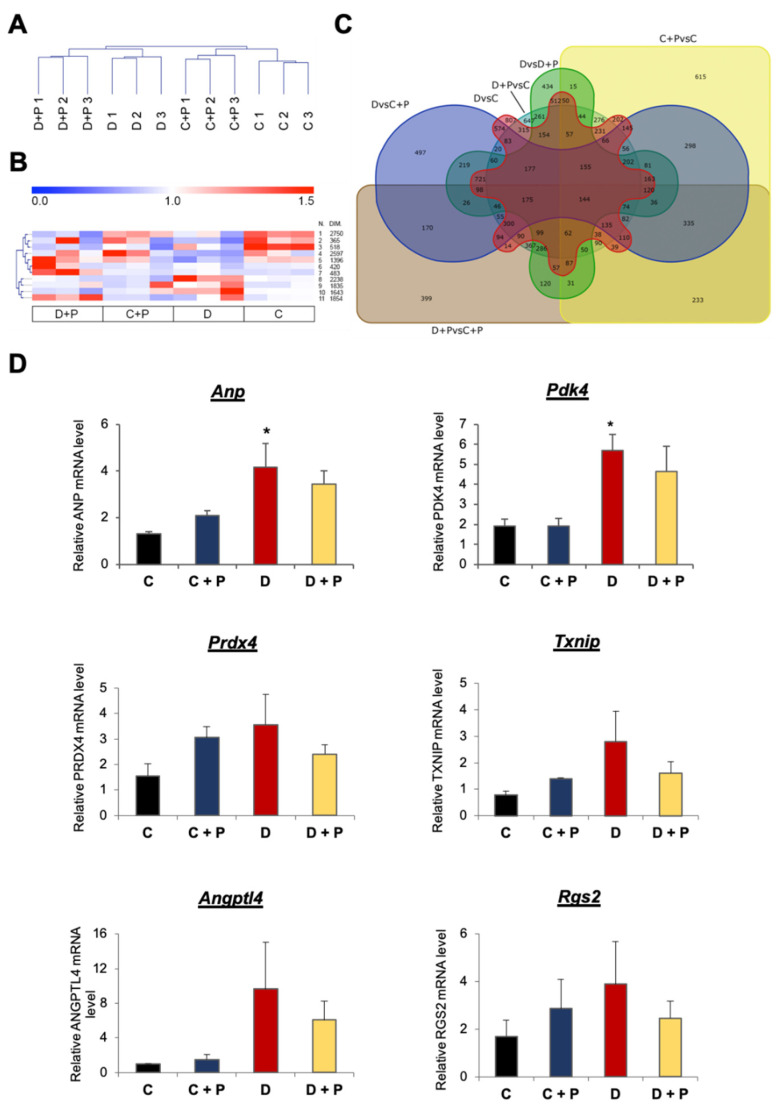
mRNA gene expression. (**A**) Dendrogram describing relationship of samples obtained according to mRNA gene expression. (**B**) Clusters of differentially expressed genes. Gene expression was calculated in relation to the average expression of the gene in all samples. (**C**) Venn diagram describing the number of differentially expressed mRNA for each comparison. (**D**) Validation of the mRNA microarray through qRT-PCR. mRNA levels were normalized to the housekeeping gene *Tbp.* * *p* ≤ 0.05 vs. C. C: control; C + P: control + pargyline; D: diabetes; D + P: diabetes + pargyline; *Anp*: atrial natriuretic peptide; *Pdk4*: pyruvate dehydrogenase kinase 4; *Prdx4*: peroxiredoxin 4; *Txnip*: thioredoxin interacting protein; *Angptl4*: angiopoietin-like 4; *Rgs2*: regulator of G-protein signaling 2.

Cluster analysis revealed that biological replicates were grouped together and that there was a clear separation between pargyline treated and non-treated samples. Samples from diabetic hearts treated with pargyline clustered near samples from diabetic mice but clearly separated (Figure 1A). In order to evaluate the effects of MAO inhibition on gene expression, genes were grouped based on their expression patterns. We identified three interesting clusters in which MAO inhibition by pargyline prevented changes in gene expression induced by diabetes without affecting the expression in control samples (C + P) (Figure 1B and Supplementary Table S3). Clusters 8 and 10 describe genes that were upregulated in diabetic hearts and restored to control levels with pargyline treatment without affecting the expression in the controls. On the contrary, cluster 5 describes genes that were downregulated in diabetic hearts and whose expression was rescued following pargyline treatment (Figure 1B). Interestingly, clusters 8 and 10 are enriched in genes encoding for proteins involved in the regulation of mitochondrial function, protein ubiquitination and autophagy or localized in the ER; cluster 5 is enriched in genes encoding for proteins involved in ubiquitination, transcription regulation, development of heart and vasculature, or localized in the Golgi (Table 1). Gene set enrichment analysis (GSEA) allowed the identification of altered pathways for each analyzed gene cluster. Table 2 shows major pathways among the twenty most significantly altered pathways identified for each cluster (for the complete list, see Supplementary Table S4). Genes whose expression was restored to control levels with pargyline treatment in comparison to the diabetic condition (clusters 8 and 10) are involved in the modulation of ubiquitin-mediated proteolysis, glycolysis and gluconeogenesis, oxidative stress, fluid shear stress and atherosclerosis, calcium cycling in cardiac cells, protein processing within the endoplasmic reticulum, phagocytosis, cell–cell interaction, mammalian target of rapamycin (mTOR) signaling, regulation of cytoskeleton and regulation of cardiomyocyte hypertrophy through miRNAs. Genes whose expression was upregulated by pargyline treatment compared to the diabetic condition (cluster 5) are involved in cellular senescence, epidermal growth factor receptor (EGFR) and insulin signaling pathways. 

We also compared differentially expressed genes considering all comparisons (Figure 1C). Six hundred and fifteen genes were specifically altered comparing controls treated with pargyline (C + P) and vehicle (C) (Figure 1C and Supplementary Table S5). Interestingly, these genes are involved in transcription regulation and encode for proteins located within the mitochondrion and in the inner mitochondrial membrane (Supplementary Table S6). On the other hand, genes specifically altered in the comparison between heart samples from diabetic mice and controls (807 genes; Figure 1C and Supplementary Table S5) are involved in the regulation of membrane transport (Golgi apparatus, endosomes and lysosomes), protein folding and degradation, and carbon metabolism (Supplementary Table S6). Therefore, present results suggest that diabetes leads to profound transcriptomic changes in T1D mouse hearts, and MAO inhibition prevents such changes affecting mitochondria, receptor-activated signaling pathways and proteolytic processes.

### 3.2. Validation of the mRNA Microarray

We first proceeded to validate changes in gene expression observed in the mRNA microarray. Validation was carried out on a series of randomly selected genes (Figure 1D), such as atrial natriuretic peptide (*Anp*), pyruvate dehydrogenase lipoamide kinase isozyme 4 (*Pdk4*), glucose transporter type 4 (*Glut4*), hypoxia-inducible factor 1α (*Hif1α*), thioredoxin-interacting protein (*Txnip*), peroxiredoxin 4 (*Prdx4*), angiopoietin-like 4 (*Angptl4*) and a regulator of G-protein signaling 2 (*Rgs2*). The qRT-PCR data showed a comparable expression pattern to the microarray results for most of the randomly selected genes, as confirmed by the Pearson correlation coefficient (r). A strong correlation (r ≥ 0.7) was observed for *Txnip* (r = 0.77), *Angptl4* (r = 0.82), *Anp* (r = 0.99), *Pdk4* (r = 0.99), *Prdx4* (r = 0.70) and *Rgs2* (r = 0.71); *Hif1α* (r = 0.57) and *Glut4* (r = 0.63) showed a trend and a moderate correlation (0.5 < r < 0.7). Among the four housekeeping genes (*Actb*, *Tbp*, *Gapdh* and *Rpl4*) we used, we showed data normalized to *Tbp* since it appeared as the most stable gene among all samples (Supplementary Figure S1).

### 3.3. MAO Inhibition Reduces Aberrant Autophagy Flux Activation in T1D Hearts

**Figure 2:**
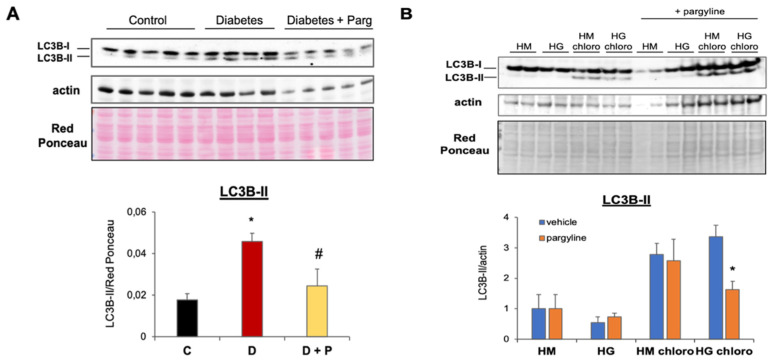
Effects of MAO inhibition on autophagy flux in T1D hearts and NRVMs cultured with high glucose. (**A**) Representative Western blot (upper panel) and densitometry analyses (lower panel) of the autophagy marker LC3B-II in control (C), diabetic (D) and diabetic mice treated with the MAO inhibitor pargyline (D + P). Values were normalized to actin or Red Ponceau staining. * *p* ≤ 0.05 vs. C; ^#^ *p* ≤ 0.05 vs. D, by Dunn’s non-parametric test. (**B**) Representative Western blots (upper panel) and densitometry analyses (lower panel) of LC3B-II in NRVMs cultured with the osmotic control mannitol (HM) or high glucose (HG) at baseline or after treatment with the inhibitor of lysosomal degradation chloroquine (chloro). Parallel experiments were performed in the presence of MAO inhibitor pargyline. Values were normalized to actin. LC3B-II abundance in HM groups was arbitrarily considered as a unit. * *p* ≤ 0.05 vs. HG chloro, with two-tailed equal variance *t*-test.

SOTA analysis revealed that clusters 8 and 10 are enriched in genes encoding for proteins involved in the proteolysis and regulation of autophagy (Table 1), suggesting that autophagy might be upregulated in diabetic hearts. In order to confirm whether processes identified by the transcriptomic and bioinformatics analyses had any biological relevance, we next assessed whether MAO activity affects autophagy flux in T1D hearts, thereby leading to mitochondrial and cellular derangements. We measured protein levels of the autophagy marker microtubule-associated protein 1 light chain 3 beta (LC3B) in cardiac tissue of T1D mice. There was a significant increase in LC3B-II in diabetic compared to control hearts (Figure 2A), suggesting that autophagy might be altered in diabetic hearts. Importantly, LC3B-II protein levels were reduced in diabetic mice treated with pargyline. 

An accurate assessment of the autophagy flux requires that all the experimental groups are paralleled by groups treated with inhibitors of lysosomal degradation. In order to evaluate changes in the autophagy flux due to T1D and/or MAO inhibition, an additional subset of experiments was performed in vitro employing NRVMs. NRVMs were cultured with high glucose in the absence or presence of MAO inhibitor pargyline and/or inhibitor of lysosomal degradation chloroquine for the last 4 h of treatment. Treatment with high glucose led to a significant upregulation of autophagy flux, while cells cultured in the presence of high glucose and pargyline displayed a dramatic reduction in autophagy, evidenced by the reduced LC3B-II accumulation following inhibition of lysosomal degradation (Figure 2B). Autophagy flux was not significantly affected by pargyline in control conditions. Therefore, these results confirm the findings obtained by the transcriptomic analyses and suggest that MAO inhibition is able to block the aberrant autophagy activation in T1D hearts.

### 3.4. MiRNA Gene Expression Analysis

**Figure 3:**
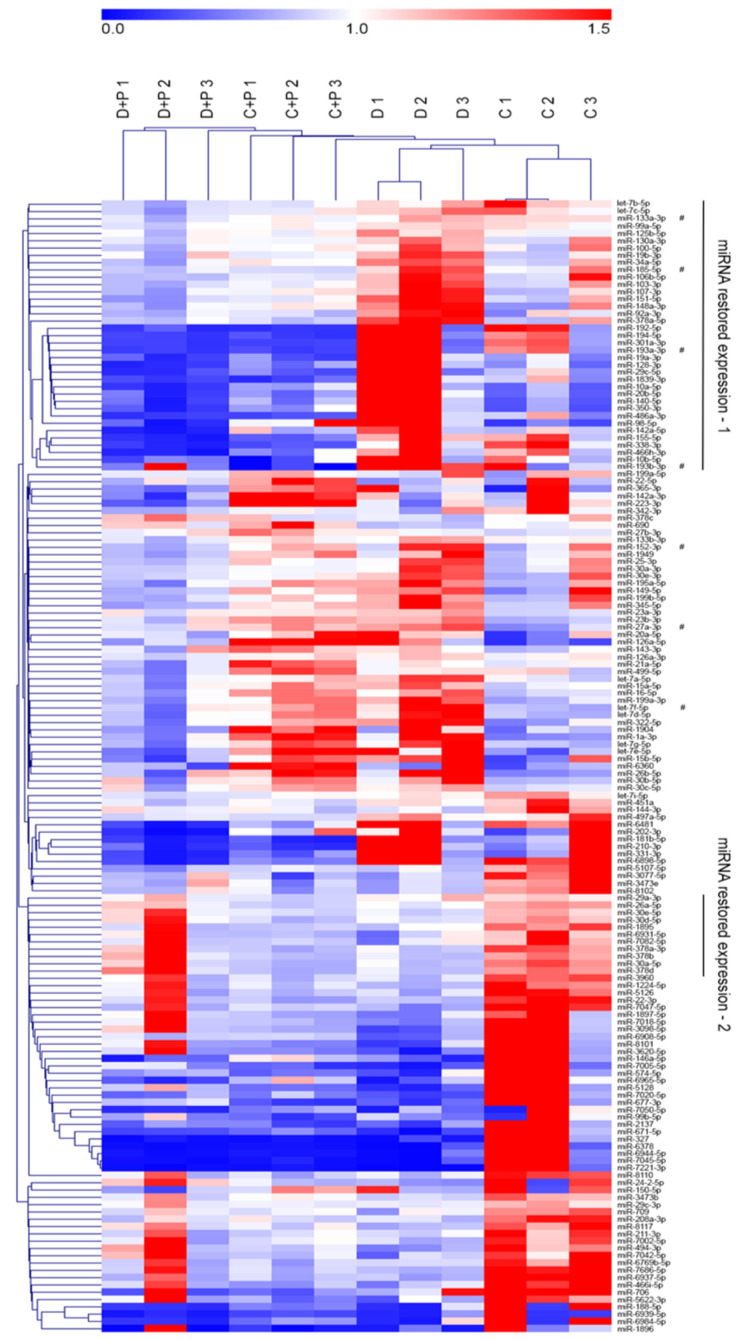
MiRNA gene expression. Heatmap of differentially expressed miRNAs. Both samples and miRNAs were clustered according to average dot products and complete linkage. Cluster 1 represents miRNAs upregulated in heart samples of diabetic mice that returned to control levels after pargyline treatment. Cluster 2 represents miRNAs downregulated in heart samples of diabetic mice that were normalized after pargyline treatment. C: control; C + P: control + pargyline; D: diabetes; D + P: diabetes + pargyline.

Regulation of cardiomyocyte hypertrophy through miRNAs was one of the pathways affected by MAO inhibition in T1D hearts (Table 2). Therefore, we next evaluated miRNA expression profiles in the same heart samples used for mRNA profiling. Sample cluster analysis did not evidence a clear separation as observed with mRNAs. Nevertheless, samples from diabetic mice and controls are completely separated from samples treated with pargyline. Moreover, among samples treated with pargyline, those derived from diabetic mice are separated from controls (Figure 3). MiRNAs were grouped in different clusters. Among these, one cluster of interest is composed of miRNAs upregulated in T1D hearts whose expression was restored to control levels by MAO inhibition (cluster 1); the second interesting cluster is composed of miRNAs that were downregulated in T1D hearts and restored to control levels with pargyline treatment (cluster 2). Importantly, the expression of miRNAs within these two clusters was unaffected in control hearts treated with pargyline (Figure 3 and Supplementary Table S7). 

In order to better characterize these clusters, we performed enrichment analysis evidencing that in cluster 1 nine miRNA families and five miRNA clusters were enriched, while in cluster 2 two miRNA families were enriched (Table 3). Anti-correlation analysis identified possible mRNA targets for miRNAs belonging to cluster 1. Interestingly, considering miRNA targets in cluster 1, the five most enriched biological processes and pathways were involved in insulin and EGFR signaling pathways, as well as cytoskeleton organization. In addition, genes regulated by miRNAs within cluster 2 are involved in the regulation of insulin signaling and autophagy (Table 4 and Supplementary Table S8 for an expanded list of genes enriched in each category). These results suggest that inhibition of MAO activity in diabetic hearts might post-transcriptionally regulate the activation of signaling pathways involved in autophagy activation and cell survival.

### 3.5. Validation of miRNA Microarray Data by qRT-PCR

**Figure 4:**
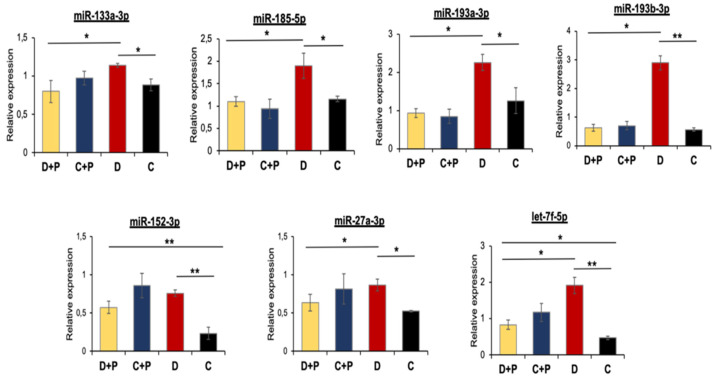
Transcriptomic miRNA expression validation. MiRNA expression was calculated through qRT-PCR relative to U6. Error bars indicate standard deviation calculated on at least three samples and three technical replicates per sample. Significance was calculated using a *t*-test between samples considering unequal variance between samples. * *p* ≤ 0.05; ** *p* ≤ 0.002. C: control; C + P: control + pargyline; D: diabetes; D + P: diabetes + pargyline.

In the same way as for mRNAs, miRNA expression was also confirmed using qRT-PCR. We chose to confirm the expression of four miRNAs falling within the cluster 1 (miR-133a-3p, -185-5p, -193a-3p, -193b-3p) and three outside of this cluster (miR-152-3p, -27a-3p, let-7f-5p) (Figure 4). In all cases, gene expression correlation between qRT-PCR and microarray was good, between 0.75 and 0.99, with miR-152-3p that showed the lowest correlation (0.5). Gene expression of all miRNAs was significantly altered comparing diabetic and control hearts (Figure 4). Interestingly, treatment of diabetic mice with pargyline prevented or reduced alterations in miRNA levels with the exception of miR-152-3p and let-7f-5p, which remained differentially expressed compared to controls (C) (Figure 4). 

**Figure 5:**
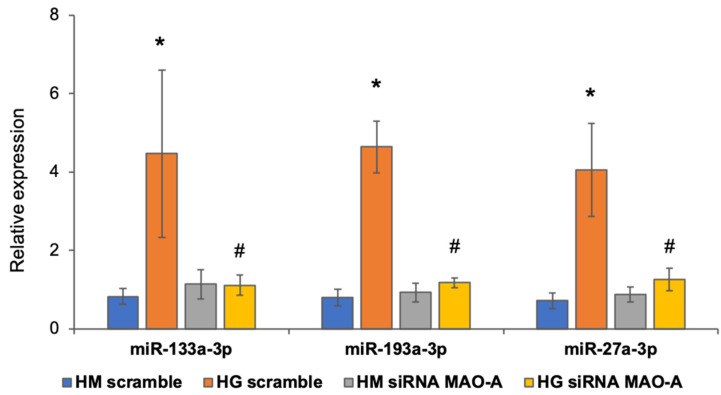
MiRNAs expression after MAO-A downregulation. MiRNAs expression was calculated through qRT-PCR relative to U6. Error bars indicate standard deviation calculated on at least three samples and three technical replicates per sample. Significance was calculated using *t*-test between samples considering unequal variance between samples. * *p* ≤ 0.05 vs. HM scramble; ^#^ *p* ≤ 0.05 vs. HG scramble.

In order to confirm that the observed effects on miRNAs expression were indeed due to MAO activity and not caused by pargyline off-target effects, we silenced MAO-A expression in NRVMs [15] and assessed expression levels of selected miRNAs after exposure of cells to high glucose. We confirmed that miR-133a-3p, miR-193a-3p and miR-27a-3p were significantly upregulated when scramble RNA-treated cardiomyocytes were exposed to high glucose, but this did not occur in NRVMs treated with siRNA against MAO-A (the main MAO isoform expressed in NRVMs) (Figure 5). This result confirms that the abovementioned miRNA expression is directly regulated by MAO-A in diabetic or high glucose conditions.

### 3.6. Function of miRNAs Affected by MAO Inhibition in T1D Hearts 

**Figure 6:**
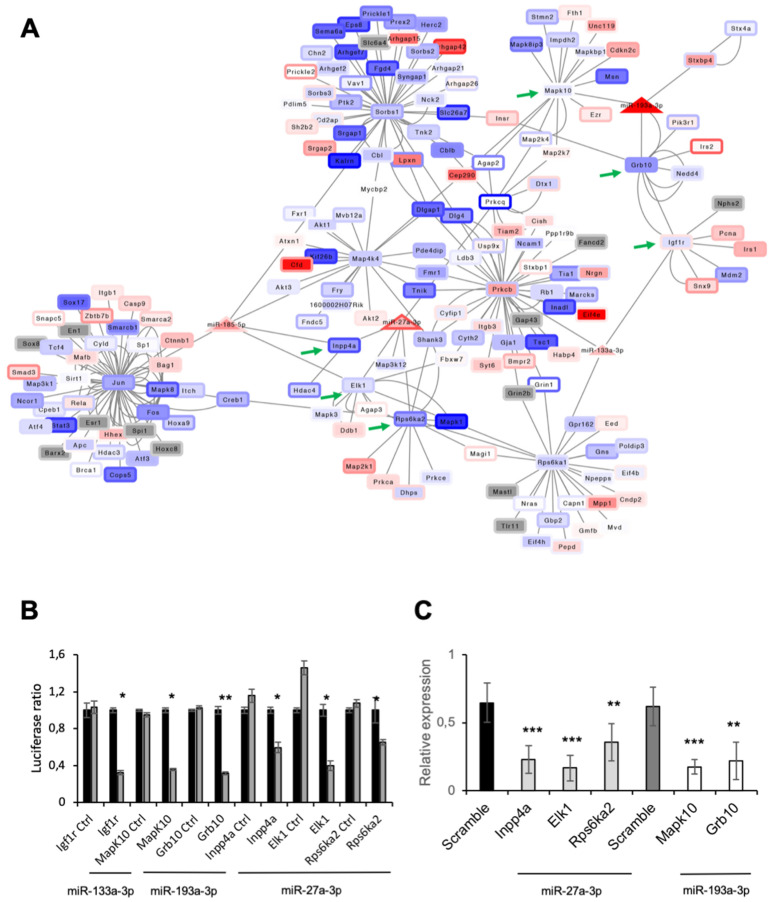
Interaction of miRNAs with their targets. (**A**) Network representing the interaction between miRNA (triangles) and targets (rectangles). Interactions among rectangles represent interactions retrieved from the BioGrid database. Colors inside nodes represent gene expression calculated as D/D + P, while border colors represent gene expression calculated as D/C. Green arrows indicate target miRNAs for which we validated miRNA–gene interactions. (**B**) Luciferase assay was performed to demonstrate the direct interaction between miR-133a-3p and *Igf1r*; miR-27a-3p and *Inpp4a*, *Elk1* and *Rps6ka2*; miR-193a-3p and *Mapk10* and *Grb10*. Part of *Igf1r*, *Inpp4a*, *Elk1*, *Rps6ka2*, *Mapk10* and *Grb10* sequences containing putative miRNA interaction sites (or not containing; Igf1r-, Inpp4a-, Elk1-, Rps6ka2-, Mapk10-, Grb10-Ctrl) were cloned into pmirGLO vector. Firefly luciferase (reporter gene) and Renilla luciferase (control reporter for normalization) activities were measured after transfection of cardiomyocytes together with miRNA mimics or a scramble sequence (Ctrl). (**C**) Gene expression of miR-27a-3p and -193a-3p targets after specific miRNA or scramble overexpression in cardiomyocytes. For both panels, data are expressed as the mean of at least four independent transfections. Significance was calculated using *t*-test between samples considering unequal variance between samples. * *p* ≤ 0.05; ** *p* ≤ 0.002; *** *p* ≤ 0.0002.

Next, we evaluated the function of miRNAs whose expression was modulated by MAO inhibition in diabetic hearts, but not in control hearts treated with pargyline (miR-133a-3p, -185-5p, -193a-3p, -193b-3p, -27a-3p). We considered their predicted targets having an expression correlation lower than -0.5 and an false discovery rate lower than 5%. No predicted targets satisfying these filters were identified for miR-193b-3p. Interestingly, predicted targets were enriched in genes involved in the organization of the cytoskeleton, formation of focal adhesions, regulation of angiogenesis and insulin-related signaling pathway (Supplementary Table S9). Regulatory networks resulting from the targets of miR-133a-3p, -185-5p, -193a-3p, -27a-3p and their interacting proteins are displayed in Figure 6A. All miRNAs had a negative expression correlation with their targets, and ratio expressions D/D + P and D/C were comparable. This confirms that pargyline treatment restored miRNA expression to control levels. 

We focused our attention on genes involved in the regulation of insulin and insulin-like growth factor 1 receptor signaling pathways to validate the interaction of miRNA–mRNA targets (Supplementary Table S9). We used luciferase assay to experimentally demonstrate the interaction between miR-133a-3p and insulin-like growth factor 1 receptor (*Igf1r*); miR-27a-3p and inositol polyphosphate-4-phosphatase type I A (*Inpp4a*), ETS transcription factor ELK1 (*Elk1*) and ribosomal protein S6 kinase A2 (*Rps6ka2*); and miR-193a-3p and mitogen-activated protein kinase 10 (*Mapk10*) and growth factor receptor bound protein 10 (*Grb10*) (Figure 6B). In order to confirm that miR-27a-3p can regulate the expression of *Inpp4a*, *Elk1* and *Rps6ka2,* and miR-193a-3p, the expression of *Mapk10* and *Grb10*, their expression was evaluated after miR-27a-3p or miR-193a-3p overexpression in cardiomyocytes. We showed that all genes were down-regulated following miR-27a-3p or miR-193a-3p overexpression (Figure 6C and Supplementary Figure S2). These results unequivocally demonstrate that MAO activity in diabetic hearts modulates levels of miR-133a-3p, -185-5p, -193a-3p, -193b-3p and -27a-3p. In addition, we showed that miR-133a-3p interacts with and controls *Igf1r* mRNA levels, while miR-27a-3p regulates levels of *Inpp4a*, *Elk1* and *Rps6ka2* mRNA, and for miR-193a-3p, that of *Mapk10* and *Grb10*. This suggests that MAO activation in diabetes might impact the activation of signaling pathways downstream of *Igf1r* via miRNA modulation.

### 3.7. MAO Inhibition Restored AKT Activation in Diabetic Hearts

**Figure 7:**
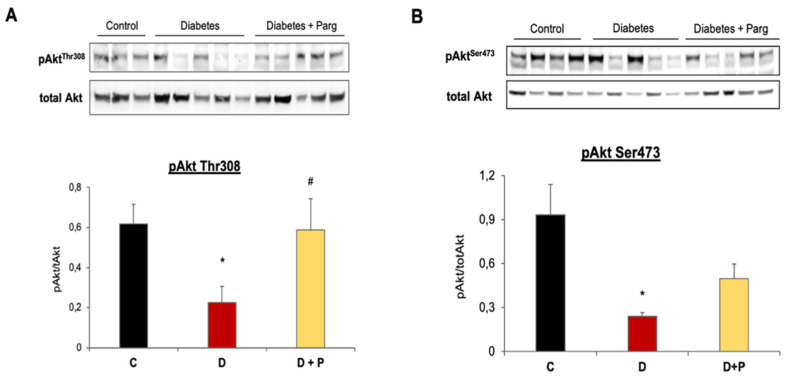
Effect of MAO inhibition on AKT activation in diabetic hearts. (**A**) Representative Western blot (upper panel) and densitometry analyses (lower panel) of the AKT phosphorylated on Thr^308^ and total AKT are shown in control (C), diabetic (D) and diabetic mice treated with the MAO inhibitor pargyline (D + P). Values were normalized to total AKT levels. * *p* ≤ 0.05 vs. C, ^#^ *p* ≤ 0.05 vs. D, with one-tailed equal variance *t*-test. (**B**) Representative Western blot (upper panel) and densitometry analyses (lower panel) of the AKT phosphorylated on Ser^473^ and total AKT are shown in control (C), diabetic (D) and diabetic mice treated with the MAO inhibitor pargyline (D + P). Values were normalized to total AKT levels. * *p* ≤ 0.05 vs. C, with one-tailed equal variance *t*-test.

The IGF1R signaling pathway is a complex network that plays a major role in cell proliferation, growth and survival. Activation of IGF1R triggers two parallel pro-survival signaling pathways via insulin receptor substrates (IRSs) and Shc [42]. IRS-1/2 phosphorylation leads to the activation of phosphatidylinositol 3-kinase/pyruvate dehydrogenase kinase 1/thymoma viral proto-oncogene 1 (PI3K/PDK1/AKT), while phosphorylation of Shc activates rat sarcoma virus (RAS), rapidly accelerated fibrosarcoma (RAF) and extracellular signal-regulated kinases/mitogen-activated protein kinases (ERK/MAPK) signaling. Therefore, in addition to *Igf1r*, we observed alterations in levels of *Grb10* and *Inpp4a*, two checkpoint molecules critical for keeping AKT activity balanced; we hypothesized that diabetes and MAO inhibition could impact the IGF1R/PI3K/AKT signaling axis downstream of the IGF1R to affect the activation of pro-survival signaling pathways. To test this, we evaluated AKT activation in cardiac tissue samples from T1D mice. We found that AKT phosphorylation on Thr^308^ and Ser^473^ was significantly reduced in T1D hearts (Figure 7), confirming the reduced activation of this signaling cascade downstream of the IGF1R. Administration of MAO inhibitor pargyline to diabetic mice prevented this reduction in AKT activation, suggesting that MAO is able to control activation of cardiomyocyte pro-survival pathways via modulation of miRNAs that eventually converge on AKT. 

## 4. Discussion

**Figure 8:**
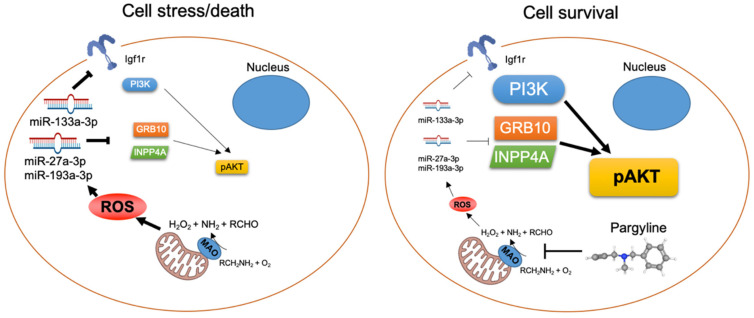
Graphical abstract of the main findings of this study indicating MAO-dependent post-transcriptional regulation of *Igf1r*, *Grb10* and *Inpp4a* via miR-133a-3p, -27a-3p and -193a-3p, which in turn regulate pro-survival signaling through AKT activation in diabetic hearts.

This study shows that MAO activity in diabetic hearts leads to profound transcriptomic changes, affecting proteolytic and pro-survival pathways activation in T1D mice in vivo. We also showed that MAOs might act as a signal leading to autophagy activation in diabetic conditions. Importantly, MAO activation in T1D hearts also targets the expression of different miRNAs, such as miR-133a-3p, -185-5p, -193a-3p, -193b-3p, -27a-3p. Among these, we confirmed *Igf1r* mRNA as the target of miR-133a-3p, *Mapk10* and *Grb10* as targets of miR-193a-3p, and identified *Inpp4a*, *Elk1* and *Rps6ka2* mRNAs as targets for miR-27a-3p. Of note, we showed that the MAO-dependent regulation of these miRNAs in diabetes impacts the activation of the IGF1R/PI3K/AKT axis (Figure 8). This provides an important link between MAO activity, transcriptomic changes, and activation of autophagy and pro-survival pathways in diabetic cardiomyopathy. 

Strong evidence provided by our previous studies highlights that mitochondrial ROS formation and, in particular, MAO-A and -B activity contributes to cardiac damage [1,9,43]. Indeed, diastolic and/or systolic dysfunction occurring in T1D mice and pressure overload is completely prevented upon MAOs inhibition [11,12,15]. We demonstrated that MAOs activation leads to an upregulation of ROS in the mitochondrial matrix [44], directly targeting the mitochondrial respiratory chain or the permeability transition pore to induce mitochondrial dysfunction [11,15]. Other studies identified MAO-A as the major isoform involved in endothelial and LV dysfunction in diabetic rat hearts using MAO-A inhibitor clorgyline [16,45]. The study by Umbarkar et al. found that diabetic hearts display a trend towards an increase in uncoupling protein 3 (UCP3) expression [16]. In our study, we did not observe any changes in *Ucp3* expression levels between control and diabetic hearts (Supplementary Table S3). In addition, rat myocardium expresses only MAO-A [46] and would thus not allow for the investigation of the effects of the other isoform (i.e., MAO-B) on the diabetic heart. Importantly, the human myocardium expresses both MAO-A and -B, and, in our studies, we addressed the contribution of both MAO isoforms to diabetes-induced alterations. Moreover, to further investigate the mechanisms underlying MAO-induced alterations in diabetic hearts, here we evaluated whether MAO activity could impact mRNA and miRNA expression profiles induced by diabetes, as well as the biological and functional significance of such changes in gene expression. Through gene expression profiling, we evaluated changes induced by diabetes in relation to MAO activity in T1D hearts and focused our attention on differentially expressed genes between control and diabetic hearts, whose expression level was normalized by MAO inhibition. Importantly, we confirmed that MAO inhibition did not affect the expression levels of other major enzymatic ROS sources or antioxidant enzymes (Supplementary Figure S3A,B). There was a slight increase in superoxide dismutase 2 (*Sod2*) and thioredoxin reductase 2 (*Txnrd2*) levels in diabetic hearts that were restored to control levels upon pargyline treatment (Supplementary Figure S3C), suggesting that MAO inhibition led to normalization of *Sod2* and *Txnrd2* levels by reducing mitochondrial ROS formation. 

Our transcriptomic data showed that processes related to protein degradation and mTOR signaling were highly affected by MAO-generated ROS in diabetic cardiomyoapthy. Indeed, ROS may act as the principal intracellular signal transducers contributing to the induction of autophagy via direct oxidation of molecules within the autophagy machinery, such as Atg4 [47], but it still remains unclear which exact ROS sources are involved. Thus, we hypothesized that MAO activity might be involved in the modulation of autophagy in diabetic hearts. We observed an increase in LC3B-II levels in T1D mice, as well as an increase in the autophagy flux in the in vitro hyperglycemia model. Changes in LC3B-II levels observed in the in vivo study are slightly different compared to the in vitro study. One possible explanation is that primary cardiomyocytes in culture behave differently compared to intact hearts in vivo. An additional explanation is that the autophagy flux occurs at a different rate in mature adult hearts vs. the more immature neonatal cardiomyocytes. Regardless of that, MAO inhibition completely prevented such changes, indicating that autophagy is affected in diabetic hearts in an MAO-dependent manner. Previous studies showed that MAO-A overexpression in cardiomyocytes and aberrant ROS formation lead to lysosomal dysfunction, autophagy impairment and heart failure [48], likely due to the non-physiological MAO-A overexpression. On the other hand, another study characterized MAO-A-generated ROS as a mediator of quality control signaling, suggesting that an increase in MAO-A protein levels leads to an upregulation of autophagy necessary for the removal of damaged macromolecules/organelles [49]. This is consistent with our observations that further extend this concept, pointing to the fact that excessive autophagy activation and/or impaired autophagosome removal leads to cardiomyocyte death and are deleterious for the diabetic heart [50]. Interestingly, Xu et al. demonstrated that T1D-induced cardiac damage was rescued upon autophagy inhibition in either beclin 1- or autophagy related 16 (Atg16)-deficient mice [51]. This reduction in the canonical autophagy pathway was associated with the activation of the non-canonical alternative autophagy, thereby maintaining normal levels of mitophagy and limiting diabetic cardiac injury [51,52,53]. Further studies are necessary to elucidate whether activation of non-canonical Ras-related protein Rab9-dependent autophagy takes place in T1D hearts upon MAO inhibition to sustain cardiac function.

Among miRNAs whose expression was restored to control levels by MAOs inhibitor, we selected miR-193a-3p, which resulted among the top ten miRNAs showing the most profound changes in the diabetic hearts following MAOs inhibition, and miR-133a-3p, -185-5p and -27a-3p resulted in the top ten showing more modest changes. By using different algorithms and biologically relevant data for miRNA target prediction, we identified *Igf1r* and several components of the signaling pathway downstream of IGF1R as putative targets for miR-133a-3p, miR-193a-3p and miR-27a-3p. These interactions were confirmed experimentally, demonstrating that miR-133a-3p directly targets *Igf1r* mRNA, whereas miR-193a-3p and miR-27a-3p target *Grb10* and *Inpp4a* mRNAs, respectively, two proteins involved in the regulation of PI3K/AKT pathway.

MiR-133a-3p is one of the most abundant miRNAs in myocyte cells, and its regulation of IGF1R expression was described previously [27,54]. ROS can control both miR-133a-3p and IGF1R expression, but the exact mechanisms, as well as ROS sources, involved in this regulation remained elusive [55,56,57]. Our results delineated a link between MAOs, ROS, miR-133a-3p and *Igf1r*, suggesting that MAO activity in diabetes leads to an upregulation of miR-133a-3p that, in turn, results in the degradation of *Igf1r* mRNA. Insulin-like growth factor-1 (IGF-1) is the ligand binding to the IGF1R that promotes the survival of cardiomyocytes and physiological hypertrophy [58,59,60,61,62]. However, the beneficial role of IGF1R was questioned by more recent studies, showing that reduced IGF1R signaling attenuated adverse cardiac remodeling in aged mice, and treatment with IGF1R monoclonal antibodies improved cardiac function in female mice [63,64]. These apparently contradictory findings can be reconciled by examining the role of IGF1R in young vs. old mice. IGF1R activation increases cardiac contractility and function in young mice, but it can be detrimental in old mice leading to an impairment in the autophagy flux and oxidative phosphorylation in the heart [65]. Regardless of age, numerous studies showed that promoting IGF1R-dependent anti-apoptotic signaling is cardioprotective in pathological conditions, such as doxorubicin-induced cardiotoxicity or myocardial infarction [66,67,68]. The present results contribute to and further extend that knowledge, indicating that *Igf1r* mRNA levels are reduced in T1D, and that restoration of IGF1R-dependent anti-apoptotic signaling via AKT obtained after administration of MAO inhibitors protects from diastolic dysfunction in diabetic hearts. 

Numerous studies demonstrated that ROS and oxidative stress interfere with the activation of the PI3K/AKT pathway [69]. It is well accepted that phosphatases, such as tyrosine phosphatases and phosphatase and tensin homolog (PTEN), can become inactivated through oxidation and thus eventually result in higher activation of the pathway [69]. However, chronic oxidative stress, in particular the alteration of the redox status observed in diabetes, results in extensive oxidative changes and nitrosylation of the components of the PI3K/AKT pathway leading to its inactivation [70]. Here we showed that there is an additional layer of regulation. We found that MAO activity, likely due to MAO-dependent ROS formation, can modulate AKT activation through post-transcriptional regulation of the IGF1R, as well as critical checkpoint molecules that keep the PI3K/AKT pathway in a homeostatic balance.

MiR-27a-3p is a redox-sensitive miRNA and is induced by oxidative stress [71]. In addition, both miR-133a-3p and -27a-3p are upregulated in failing diabetic hearts and are involved in the regulation of pro-hypertrophic signaling, autophagy and oxidative stress regulation [72,73]. Here, we confirmed these findings and identified *Inpp4a* as the direct target of miR-27a-3p. INPP4A is a lipid phosphatase that specifically dephosphorylates phosphatidylinositol (3,4)-bisphosphate and acts as a negative regulator of the PI3K/AKT pathway [74]. In the central nervous system, INPP4A is a suppressor of glutamate excitotoxicity and neuronal cell death [75]. On the other hand, INPP4A overexpression in fibroblasts impairs fibroblast proliferation and growth [74]. Although the role of INPP4A in the heart has not been investigated to date, the present results suggest that INPP4A downregulation in the diabetic myocardium likely contributes to cardiomyocyte apoptosis. Nevertheless, it cannot be excluded that this downregulation may occur as compensation in response to the reduced activation of the IGF1R-dependent signaling cascade. 

In the present study, we found that *Grb10*, an additional negative regulator of the PI3K/AKT pathway, was also downregulated in diabetic hearts. In this regard, we identified miR-193a-3p as the miRNA that directly binds to the 3′-UTR of *Grb10* mRNA leading to its degradation. miR-193a-3p is controlled by ROS and is upregulated in myocardial infarction, thereby promoting cardiomyocyte apoptosis [76,77]. Activated IGF1R can epigenetically silence miR-193a-3p through activation of the PI3K/AKT/DNA methyltransferase (cytosine-5) 1 (DNMT) signaling pathway [78]. Whether downregulation of IGF1R-mediated signaling in diabetic hearts leads to an increase in miR-193a-3p levels remains to be fully elucidated. 

In summary, our results indicate that diabetes leads to profound transcriptomic changes in mouse hearts and that MAO inhibition prevents such changes. We identified autophagy as one of the biological processes affected in an MAO-dependent manner. Finally, we described, for the first time, a subset of miRNAs whose expression is directly regulated by MAOs. These miRNAs, namely miR-133a-3p, miR-27a-3p and miR-193a-3p, are involved in the regulation of signaling pathways that eventually converge on the regulation of AKT activity to promote pro-survival signaling. Of note, MAO activity is increased in failing human LVs and in atrial appendages from patients with atrial fibrillation [79,80]. In addition, a recent study found that MAO-A and MAO-B expression and activity were higher in the atrial myocardium of patients with type 2 diabetes [81]. Therefore, targeting MAOs may prove to be beneficial in patients with diabetes and cardiac disease. Future studies should aim at assessing whether MAO-dependent modulation of mRNA and miRNA expression profiles also occurs in failing human hearts. 

## Figures and Tables

**Table 1:** Summary of enrichment scores for gene clusters identified by SOTA algorithm. Data described in the table were retrieved using the DAVID database. GO indicates the Gene Ontology number, while KW stands for keywords used in the UniProt database.

Cluster Number	Description	*p*-Value
8	GO:0005783 Endoplasmic reticulum	0.000027
	KW-0496 Mitochondrion	0.017
	GO:0097352 Autophagosome maturation	0.005
10	KW-0832 Ubl conjugation	0.00049
	KW-0496 Mitochondrion	0.000017
	GO:0005783 Endoplasmic reticulum	0.00087
	GO:0004842 Ubiquitin-protein transferase activity	0.02
5	KW-0832 Ubl conjugation	1.9 × 10^−8^
	GO:0005794 Golgi apparatus	0.0000012
	KW-0805 Transcription regulation	4.07 × 10^−6^
	GO:0003281 Ventricular septum development; GO:0060976 Coronary vasculature development	0.03

**Table 2:** GSEA results. KEGG and Wiki pathways were used to identify altered processes.

Cluster Number	Pathway	*p*-Value
	KEGG Ubiquitin-mediated proteolysis	1.74 × 10^−8^
	KEGG Fluid shear stress and atherosclerosis	4.13 × 10^−6^
	WP Oxidative Stress and Redox Pathway	8.47 × 10^−6^
	KEGG Peroxisome	0.000029
	WP Glycolysis and Gluconeogenesis	0.00010
	WP Calcium Regulation in the Cardiac Cell	0.00010
10	KEGG Protein processing in endoplasmic reticulum	2.57 × 10^-15^
	KEGG Phagosome	3.14 × 10^−11^
	KEGG Ubiquitin-mediated proteolysis	3.60 × 10^−7^
	KEGG Tight junction	4.90 × 10^−7^
	WP Calcium Regulation in the Cardiac Cell	6.80 × 10^−7^
	KEGG mTOR signaling pathway	8.04 × 10^−7^
	WP MicroRNAs in Cardiomyocyte Hypertrophy	1.10 × 10^−6^
	WP Regulation of Actin Cytoskeleton	2.92 × 10^−6^
	KEGG Peroxisome	4.77 × 10^−6^
5	KEGG Cellular senescence	2.69 × 10^−10^
	WP EGFR1 Signaling Pathway	6.92 × 10^−10^
	WP Insulin Signaling	1.14 × 10^−6^

**Table 3:** MiRNA enrichment analysis. MiRNA expression cluster indicates the correspondence of the cluster indicated in Figure 3; FDR is for false discovery rate. The column “miRNAs” shows a list of miRNAs that fall within the term and whose expression is recovered with pargyline treatment.

miRNA Expression Cluster	Term	FDR	miRNAs
1	Cluster		
	miR-17	1.54 × 10^−3^	miR-19a, miR-19b-1, miR-92a-1
	miR-106a	1.54 × 10^−3^	miR-20b, miR-19b-2, miR-92a-2
	miR-99a	3.71 × 10^−3^	miR-99a, let-7c
	miR-100	9.10 × 10^−3^	miR-100, miR-125b-1
	miR-6749	0.0163	miR-194-2, miR-192
	Family		
	miR-10	5.71 × 10^−8^	miR-100, miR-10a, miR-10b, miR-125b-1, miR-125b-2, miR-99a
	miR-19	1.18 × 10^−4^	miR-19a, miR-19b-1, miR-19b-2
	miR-194	3.71 × 10^−3^	miR-194-1, miR-194-2
	miR-193	3.71 × 10^−3^	miR-193a, miR-193b
	miR-128	3.71 × 10^−3^	miR-128-1, miR-128-2
	miR-133	9.10 × 10^−3^	miR-133a-1, miR-133a-2
	let-7	0.0109	let-7b, let-7c, miR-98
	miR-130	0.0163	miR-130a, miR-301a
	miR-25	0.0163	miR-92a-1, miR-92a-2
2	Family		
	miR-30	1.66 × 10^−4^	miR-30a, miR-30d, miR-30e
	miR-378	1.25 × 10^−7^	miR-378a, miR-378b, miR-378d

**Table 4:** MiRNA target enrichment analysis. Wiki pathways (WP) and biological processes (BP) enriched among the miRNA targets.

miRNA Expression Cluster	Pathway	*p*-Value	Overlap Genes
1	WP Insulin Signaling	3.95 × 10^−19^	JUN, SNAP25, MAP2K2, IGF1R, INPP4A, SNAP23, PIK3CA, PIK3CD, CBLB, MAP3K8, SORBS1, FLOT2, MAPK4, MAP3K7, GRB10, MAP4K4, CRK, PIK3R3, RPS6KA2, RPS6KA1, MINK1, GSK3B, RAF1, MAPK12, SLC2A4
	WP EGFR1 Signaling	7.72 × 10^−17^	JUN, WNK1, MAP2K2, PIK3CA, PIK3CD, CBLB, ASAP1, WASL, STAT3, STAT1, VAV3, GJA1, CTNND1, CAV1, CREB1, HTT, GRB10, EPS15, CRK, PIK3R3, RALBP1, RPS6KA2, RPS6KA1, RAF1
	WP Regulation of Actin Cytoskeleton	3.45 × 10^−15^	MAP2K2, MSN, SSH2, SSH1, ARHGEF7, PIK3CA, LIMK1, PIK3CD, ITGA1, ARHGEF6, MAPK4, APC, MYLK, PAK2, IQGAP1, CRK, WASF2, PIK3R3, ARPC5, PTK2, RAF1
	BP actin cytoskeleton organization	3.52 × 10^−16^	ABLIM3, ACTR2, ARFIP2, ARHGEF18, ATXN3, CDC42BPA, CORO1C, CORO2B, CRK, CSRP1, FLNB, PIK3CA, SDAD1, SSH1, SSH2, TAOK2, WASF2, WASL, ACTN4, LIMK1, SPTAN1, DMD, UTRN, SPTBN1
	BP endocytosis	4.66 × 10^−15^	AAK1, ANK2, ANKFY1, AP2A2, CAV1, CLCN5, DNM1L, DNM2, DNM3, FCHSD2, FKBP15, FNBP1L, MICALL1, MYO6, PSTPIP1, RABEP2, RALBP1, SGIP1, SYNRG, WASF2, NCKIPSD, ITSN1, EPS15
2	WP Insulin Signaling	0.005	PRKAA2, CBLB, PRKCA, RPS6KB1
	BP negative regulation of insulin receptor signaling pathway	0.016	PRKCA, PTPN2, RPS6KB1
	BP positive regulation of autophagy	0.04	MTDH, PRKAA2, DEPDC5

## Data Availability

Microarray data were submitted to the GEO database (SuperSeries GSE210612 is composed of miRNA data (GSE210036) and mRNA data (GSE210611)).

## Supplementary Materials

The following supporting information ca be downloaded at https://www.mdpi.com/article/10.3390/cells11172697/s1. Supplementary Figure S1. Evaluation of the expression stability of housekeeping genes for qRT-PCR analyses. Supplementary Figure S2. MiR-27a-3p and -193a-3p levels following their overexpression. Supplementary Figure S3. (A) Gene expression of enzymes involved in ROS production, i.e., NADPH oxidase 1 (*Nox1*), NADPH oxidase 4 (*Nox4*) and myeloperoxidase (*Mpo*). (B) Gene expression of antioxidant enzymes catalase (*Cat*), glutathione peroxidase 3 (*Gpx3*), glutathione peroxidase 7 (*Gpx7*), peroxiredoxin 1 (*Prdx1*) and peroxiredoxin 6 (*Prdx6*). (C) Relative expression of superoxide dismutase 2 (*Sod2*), thioredoxin reductase 2 (*Txnrd2*) and peroxiredoxin 4 (*Prdx4*). * *p* < 0.05 vs. C, ^#^ *p* < 0.05 vs. D. Supplementary Table S1. List of primers used for the qRT-PCRs. Supplementary Table S2. List of primers used to clone binding sites of mRNAs targeted by miRNAs and respective controls. Supplementary Table S3. Description of genes within each cluster described in Figure 1B. Supplementary Table S4. Gene set enrichment analysis for clusters 5, 8 and 10 described in Figure 1B. Supplementary Table S5. Differentially expressed genes. Supplementary Table S6. Enrichment analysis of 615 genes found to be specifically altered comparing controls treated with pargyline (C + P) and vehicle (C). Supplementary Table S7. Expression of miRNAs belonging to clusters 1 and 2 represented in Figure 3. Supplementary Table S8. Function of genes targeted by miRNAs belonging to clusters 1 and 2. Supplementary Table S9. Functional analyses for predicted targets of miR-133a-3p, -185-5p, -193a-3p, -193b-3p, -27a-3p.
