# Supplementary material for: Monoamine Oxidase-Dependent Pro-Survival Signaling in Diabetic Hearts Is Mediated by miRNAs
Source: Cells. 2022 Aug 30;11(17):2697. doi: 10.3390/cells11172697 (PMC9454570; doi:10.3390/cells11172697)
Supplement: Supplementary file 1 [file cells-11-02697-s001.zip › Supplementary Figures final-.pdf]

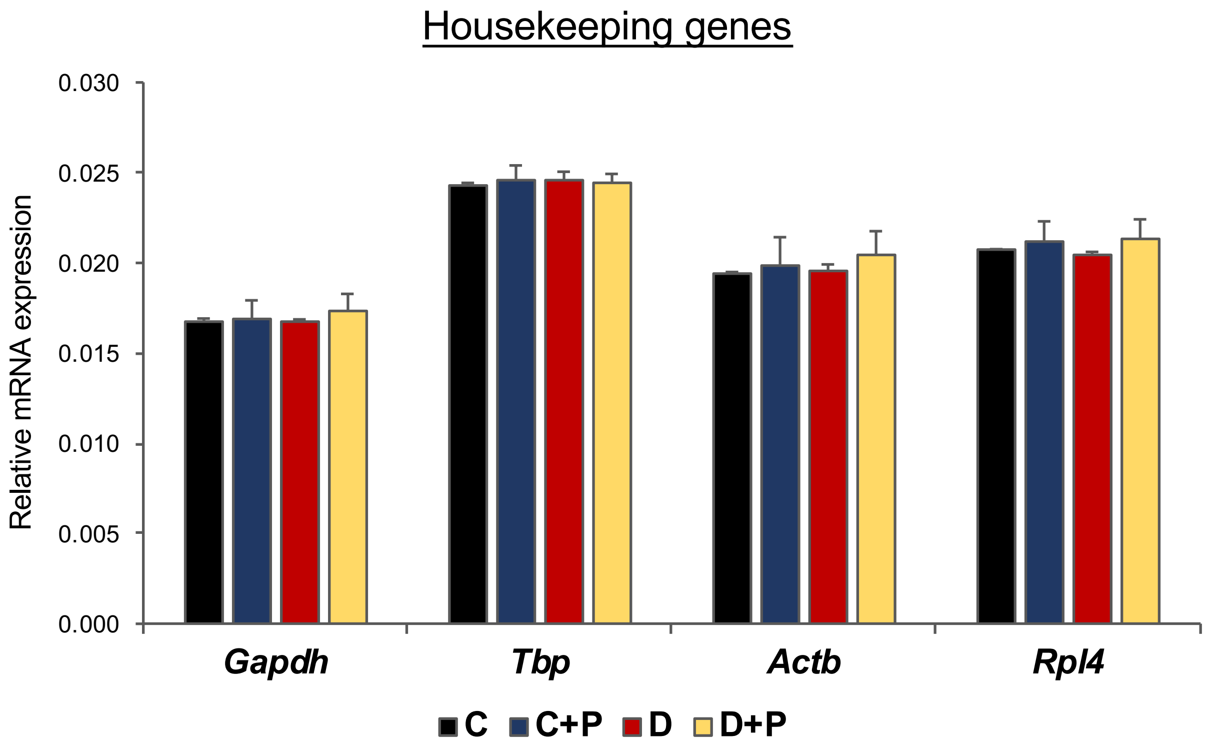

Supplementary Figure S1. Evaluation of the expression stability of housekeeping genes for qRT-PCR analyses.

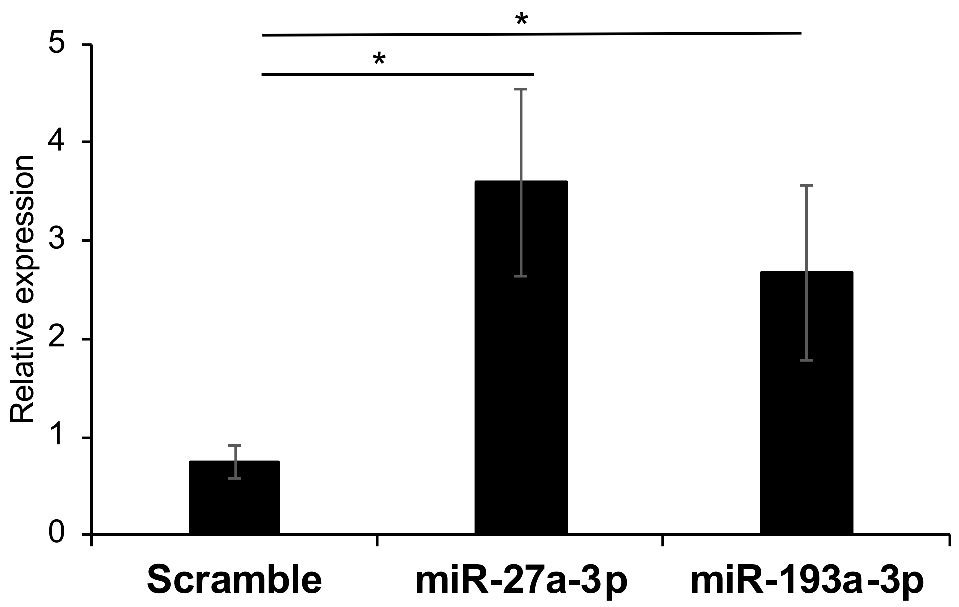

Supplementary Figure S2. MiR-27a-3p and -193a-3p levels following their overexpression. *p<0.05 vs Scramble.

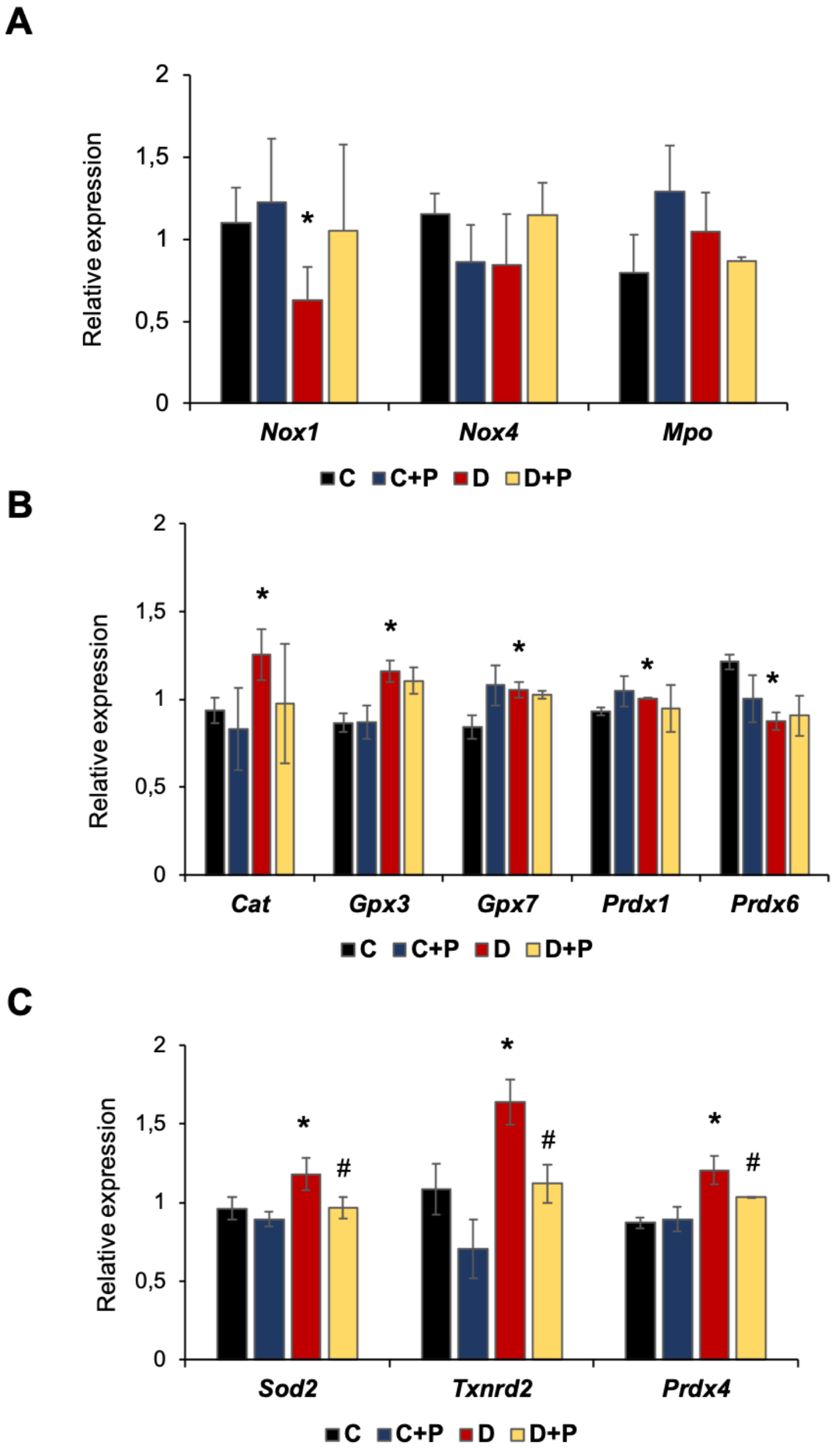

Supplementary Figure S3. (**A**). Gene expression of enzymes involved in ROS production, i.e. NADPH oxidase 1 (*Nox1*), NADPH oxidase 4 (*Nox4*), and myeloperoxidase (*Mpo*). (**B**). Gene expression of antioxidant enzymes catalase (*Cat*), glutathione peroxidase 3 (*Gpx3*), glutathione peroxidase 7 (*Gpx7*), peroxiredoxin 1 (*Prdx1*) and peroxiredoxin 6 (*Prdx6*). (**C**). Relative expression of superoxide dismutase 2 (*Sod2*), thioredoxin reductase 2 (*Txnrd2*) and peroxiredoxin 4 (*Prdx4*). $^{*}p \leq 0.05$ vs C, $^{\#}p \leq 0.05$ vs D.
